# Nitrogen-Passivated germanium carbide nanomeshes as potential catalysts for photocatalytic water splitting

**DOI:** 10.1038/s41598-025-11711-6

**Published:** 2025-07-20

**Authors:** Sarah Gamal, Ghada E. Khedr, M. Nashaat, Lobna M. Salah, Ahmed A. Maarouf, Nageh K. Allam

**Affiliations:** 1 Department of Physics, Faculty of Science, Cairo University, Cairo, 12613 Egypt; 2 Department of Analysis and Evaluation, Egyptian Petroleum Research Institute, Cairo, 11727 Egypt; 3 Department of Physics, Faculty of Basic Sciences, The German University in Cairo, New Cairo, 13411 Egypt; 4 Energy Materials Laboratory, School of Sciences and Engineering, Physics Department, The American University in Cairo, New Cairo, 11835 Egypt

**Keywords:** g-GeC, 2D materials, OER, HER, Water splitting, DFT, Energy science and technology, Materials science, Nanoscience and technology

## Abstract

Nitrogen-passivated germanium carbide (GeC) nanomeshes have been systematically investigated as efficient photocatalysts for water splitting. The nanomesh, characterized by a lattice constant of 19.3 Å and a pore diameter of 7.3 Å, maintains a planar architecture with optimized N-Ge and N-C bond lengths of 1.8 Å and 1.3 Å, respectively. Partial density of states (PDOS) analysis indicates that the conduction band is predominantly governed by Ge states, while C states dominate the valence band. Nitrogen incorporation critically alters the electronic structure near the band edges, significantly influencing photocatalytic behavior. Notably, introducing porosity reduces the bandgap from 2.04 eV (pristine GeC) to 1.33 eV in the N-passivated configuration. The calculated band edge positions straddle the redox potentials of water, indicating thermodynamic feasibility for overall water splitting. Several favorable sites were identified for the hydrogen evolution reaction (HER), with nearly thermoneutral ΔG values, suggesting high catalytic efficiency. For the oxygen evolution reaction (OER), the formation of OH* was determined to be the rate-limiting step with a ΔG_1_ = 1.84 eV. Bader charge analysis confirmed electron transfer from the OH* species to the adjacent Ge atom, resulting in a net gain of 0.39 |e| by Ge. These findings demonstrate that N-passivated GeC nanomeshes exhibit a favorable electronic structure and catalytic surface characteristics for photocatalytic water splitting.

## Introduction

The global demand for sustainable and clean energy continues to rise in response to population growth and environmental concerns^1-3^. Hydrogen has emerged as a highly promising alternative energy carrier due to its high energy density and zero carbon emissions upon combustion. One of the most attractive routes for hydrogen generation is photocatalytic water splitting, which utilizes water, a clean, abundant resource, driven by sunlight.

Two-dimensional (2D) materials have attracted extensive interest for photocatalytic applications because their ultrathin nature enhances charge carrier separation and minimizes recombination^4-5^. Density functional theory (DFT) investigations have played a crucial role in screening and understanding the catalytic potential of various 2D systems. For instance, pristine and functionalized WS₂ and PtS_2_ nanosheets have been explored, with Pd-doped WS₂ showing optimal hydrogen binding energy for the hydrogen evolution reaction (HER)^6^. Likewise, carbon-doped 2D boron sheets displayed strong catalytic activity for both HER and oxygen evolution reaction (OER)^7^. Te-doped TiO₂, particularly with Te substitution and hydrogen interstitials (TeTi + H_i_), emerged as a promising HER catalyst, while TeTi + O_v_ configurations were superior for OER^8^. Experimentally, CdS has been validated as a viable photocatalyst, owing to its suitable band edge alignment with water redox potentials^9^.

Recently, 2D transition metal carbides have gained traction due to their excellent electrical conductivity, tunable bandgaps, and catalytic activity^10^. Among these, monolayers such as NbC_2_, TaC_2_, and MoC_2_ have demonstrated bifunctional electrocatalytic behavior. TaC_2_, for example, exhibited low overpotentials (0.06 V for HER and 0.37 V for ORR), while MoC_2_ achieved superior performance in overall water splitting (0.001 V for HER and 0.45 V for OER), underscoring the potential of 2D MC₂ systems. Graphitic carbon nitride (g-C₃N₄) is another prominent material studied extensively for water splitting. Ali et al. reviewed advances in its chemical modifications—such as intercalation, doping, and nanostructuring, which significantly improved its hydrogen generation performance^11^. Modified g-C₃N₄-based quantum dots exhibited exceptional photocatalytic durability over multiple cycles. Both 1D and 2D morphologies of g-C₃N₄ have proven effective in hydrogen production and broader green chemistry applications.

Innovative computational approaches are also emerging. Zheng et al. developed a deep learning model using gated recurrent units (GRUs) trained on multifactorial data to predict hydrogen production kinetics under simulated sunlight^12^. The model identified radiation intensity as the most influential factor in photocatalytic activity, offering a robust framework for scaling up hydrogen production. On the experimental front, novel materials such as Fe_7_S_8_/C electrocatalysts synthesized via co-precipitation have shown excellent HER activity across the full pH range^13^. Pt-doped TiO₂ hollow spheres have also demonstrated outstanding hydrogen evolution performance, achieving a rate of 1023.71 µmol·h⁻¹·g⁻¹ at room temperature^14-15^. Furthermore, transition-metal doping in dioxaporphyrin cavities revealed optimized ΔG values for hydrogen adsorption, with Fe and Mn dopants outperforming even Pt^16^. Recent studies on V-Cu_2_S electrodes for alkaline water electrolysis also show promise, achieving current densities of 100 mA/cm² at overpotentials of 375 mV (HER) and 403 mV (OER). When configured into a bifunctional electrolyzer, these catalysts operated efficiently at 1.97 V^17^.

Among the newer classes of materials, 2D graphitic germanium carbide (g-GeC) stands out due to its direct bandgap of 2.26 eV, stable honeycomb lattice, and favorable electronic properties stemming from delocalized *pz* orbitals^18-19^. Though it exhibits an indirect bandgap in its pristine form, several DFT studies have highlighted its potential for photocatalytic water splitting. For instance, GeC/SiC heterostructures under biaxial strain showed significant activity under UV and visible light, while BAs/GeC bilayers exhibited similar enhancements^20-21^. Surface passivation of GeC with various atoms (e.g., F, P, Se) has led to significant improvements in solar-to-hydrogen (STH) conversion efficiency, with F-GeC-SeF reaching 20.74% under 3% tensile strain^22^.

A powerful approach for tuning the electronic properties of 2D materials involves introducing periodic pores^23^. In honeycomb structures like g-GeC, such nanomeshes not only modify bandgaps but also introduce additional degrees of freedom for electrons, including valley pseudospin^24^. Porous g-GeC has previously been reported to maintain structural stability while exhibiting a tunable bandgap depending on the nature of edge passivation, ranging from 1.18 to 1.93 eV^25^.

In this work, we explore a novel porous nitrogen-passivated GeC nanomesh as a photocatalyst for water splitting. The nanomesh, with a lattice constant of 19.3 Å and a bandgap of 1.33 eV, features 12 potential adsorption sites that were fully optimized to evaluate their HER and OER activities. To our knowledge, this is the first comprehensive study of nitrogen-passivated GeC nanomeshes, combining electronic structure analysis, reaction energetics, and charge transfer behavior. Our findings highlight the material’s promise as a next-generation photocatalyst with optimal electronic properties and active surface sites.

## Computational methods

Various adsorption sites were examined for HER utilizing the Quantum Espresso plane-wave density functional theory program^26^. Furthermore, the GGA, which is part of the Perdew-Burke-Ernzerhof (PBE) framework, is used as the exchange-correlation functional with an energy cutoff of 45 Ry and a force threshold of 10⁻³ eV/Å. To separate images in a direction perpendicular to the nanomesh’s plane, a vacuum distance of 12 is used. Van der Waals interactions (vdW) were calculated within the semi-empirical DFT-D^27^ and the first-principles vdW-DF methods^28,29^.The OER calculations were carried out utilizing the Vienna Ab-Initio Simulation Package (VASP), which applies spin-polarized DFT. This approach was used to compute the Gibbs free energies of the slab and its intermediate states. The PBE exchange-correlation functional, widely employed within the GGA, was applied. With an energy convergence threshold of 10^−8^ eV and an energy cutoff of 500 eV, the calculations were carried out. A 4 × 4 × 1 k-point mesh was utilized, and Bader charge analysis was performed following the method developed by Henkelman et al.^30^. Molecular dynamics based on ab initio simulations were employed inside a canonical ensemble (NVT) with a 2.0 fs time step to examine the temperature stability of the catalyst under study. We used the Nosé-Hoover chain thermostat to keep the temperature constant and ran the simulations for a total of 10 ps.

## Results and discussion

**Fig. 1:**
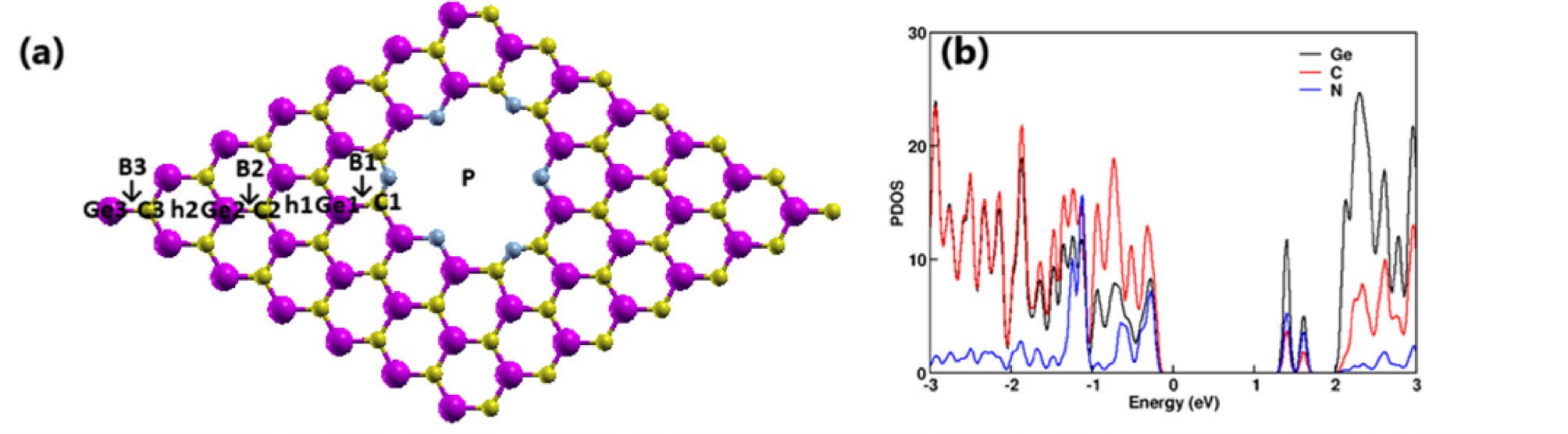
(**a**) Optimized adsorption sites for H atoms on N-passivated GeC nanomesh, and (**b**) PDOS of the N-passivated GeC nanomesh showing orbital contributions from Ge(black), C (red), and N (blue) atoms.

A nitrogen-passivated GeC nanomesh was designed by introducing a pore whose perimeter is terminated with six nitrogen atoms. The optimized structure, shown in Fig. 1a, exhibits a lattice constant of 19.3 Å and a pore diameter of 7.3 Å, maintaining a nearly planar geometry with a negligible out-of-plane deviation (~ 0.01 Å). The bond lengths for N–Ge and N–C were calculated to be approximately 1.8 Å and 1.3 Å, respectively. The electronic structure, analyzed through partial density of states (PDOS) and presented in Fig. 1b, reveals that Ge atoms primarily contribute to the conduction band, while C atoms dominate the valence band. Notably, nitrogen atoms introduce localized states near the band edges, significantly modifying the electronic environment and playing a crucial role in tuning the material’s photocatalytic properties. To further explore the water splitting capability of the nanomesh, surface adsorption energies were computed for hydrogen evolution reaction (HER) and oxygen evolution reaction (OER) intermediates. Twelve distinct adsorption sites were identified and are labeled in Fig. 1. These include the central pore site (P), three atop Ge sites (Ge_1_, Ge_2_, Ge_3_), three atop C sites (C_1_, C_2_, C_3_), two Ge–C bridge sites (B_1_, B_2_), and two hollow hexagonal sites (h_1_, h_2_). Each site was geometrically optimized and analyzed to determine its catalytic suitability for HER and OER processes.

Introducing a pore into the GeC nanosheet significantly reduces its bandgap from 2.04 eV in the pristine form to 1.33 eV in the nitrogen-passivated configuration. While bandgap narrowing is a crucial step toward enhancing photocatalytic activity, it alone is insufficient for ensuring effective hydrogen production via water splitting. For a material to function as an efficient photocatalyst, its band edges must appropriately align with the water redox potentials, specifically, the hydrogen evolution potential (H⁺/H₂) and the oxygen evolution potential (O₂/H₂O).

**Fig. 2:**
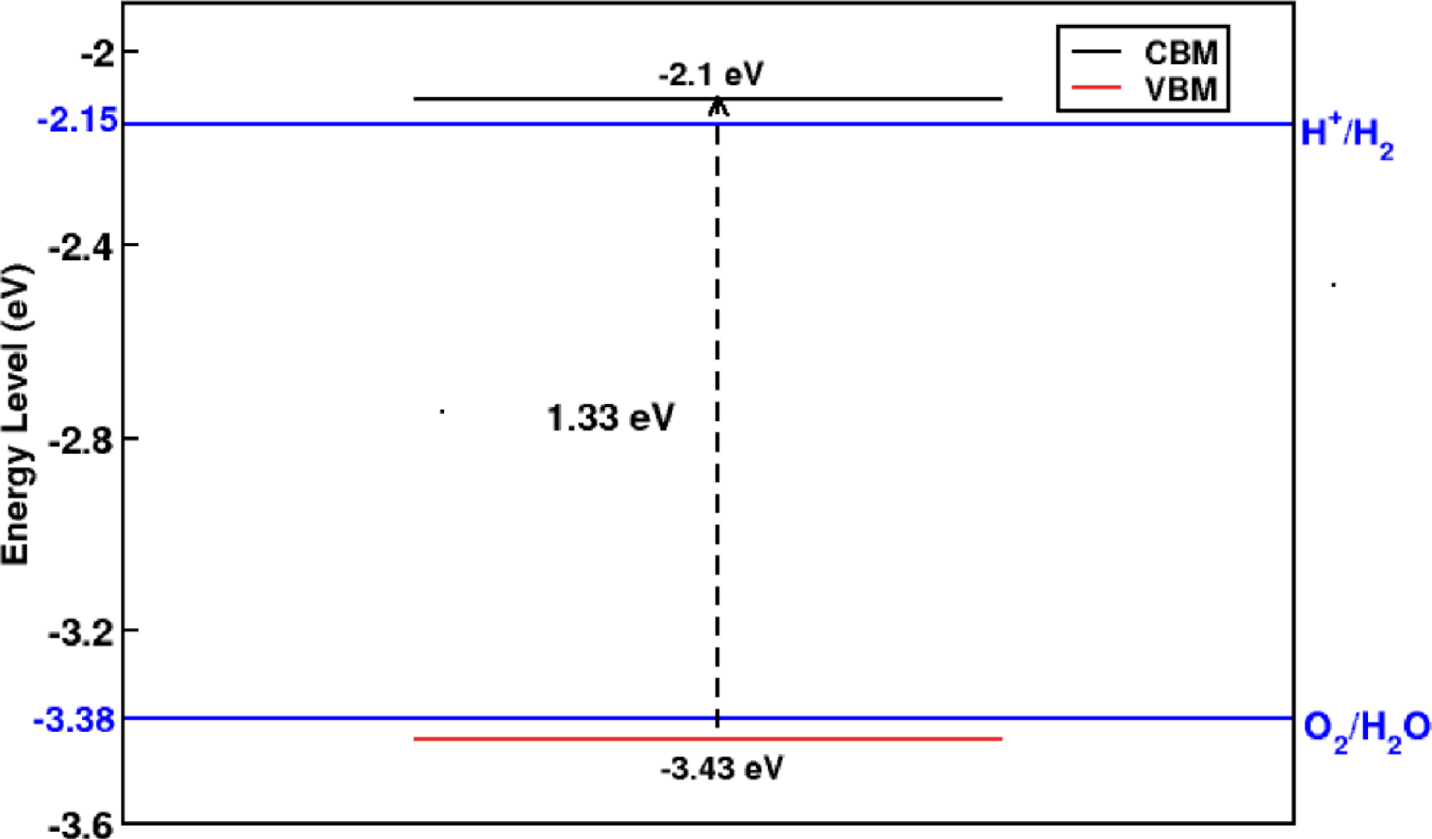
Band edge alignment of N-passivated GeC nanomesh relative to water redox potentials, indicating suitability for water splitting reactions.

As shown in Fig. 2, the conduction band minimum (CBM) of the N-passivated GeC nanomesh lies 0.05 eV above the H⁺/H₂ reduction potential, while the valence band maximum (VBM) is positioned 0.05 eV below the O₂/H₂O oxidation potential. These values are referenced to the vacuum level, which was determined using the planar-averaged electrostatic potential. This alignment confirms that both photogenerated electrons and holes possess sufficient energy to drive the overall water splitting reaction. Furthermore, the PDOS analysis in Fig. 1b indicates the absence of any localized midgap states, ensuring efficient charge transport and minimizing electron–hole recombination. Together, these results highlight the suitability of the N-passivated GeC nanomesh for photocatalytic water splitting applications.

### Hydrogen evolution reaction (HER)

**Fig. 3:**
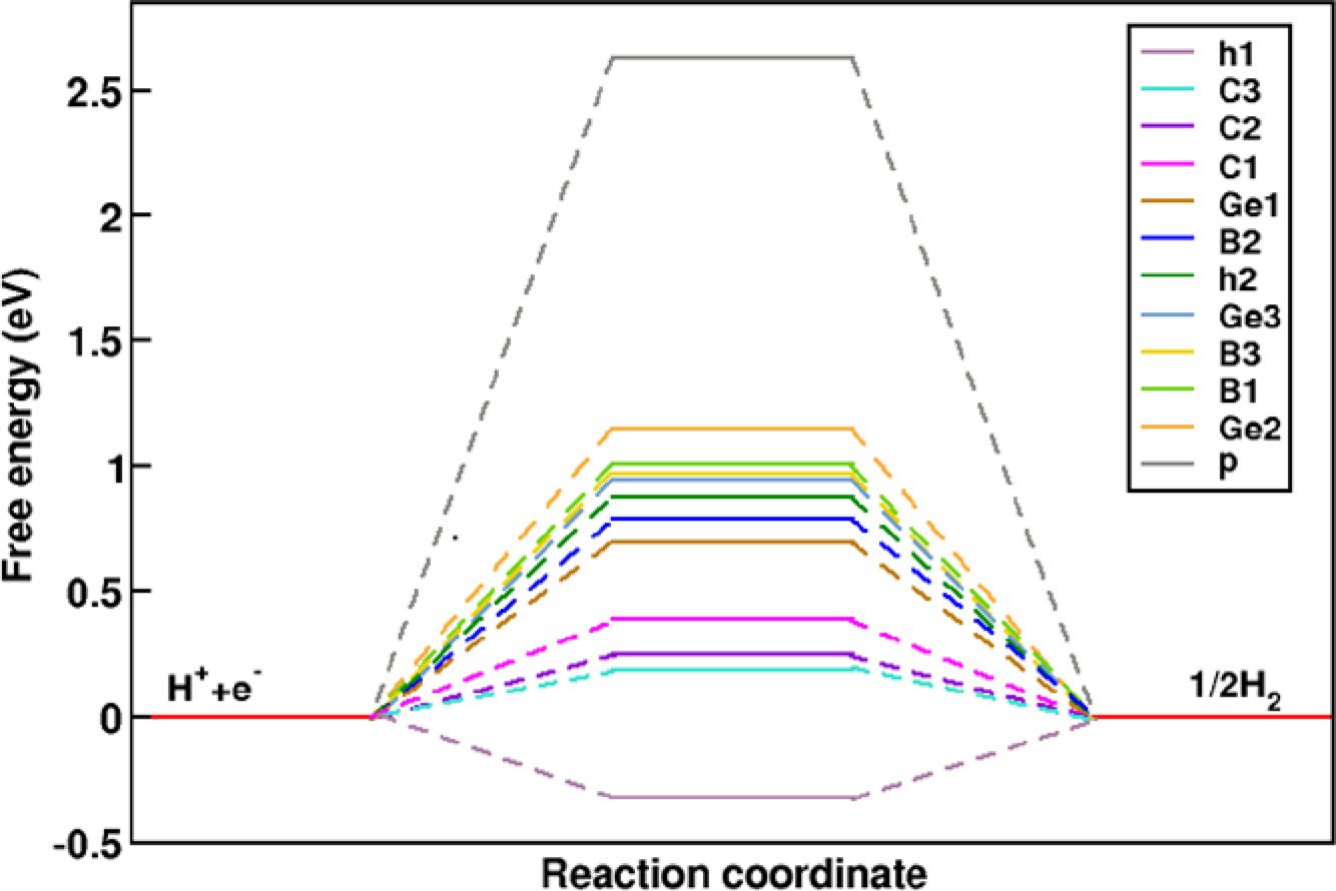
Gibbs free energy changes (ΔG) of 12 intermediate adsorption sites during the hydrogen evolution reaction (HER) on the studied surface.

The hydrogen evolution reaction (HER) proceeds through a multi-step mechanism comprising: (1) the adsorption of a water molecule, (2) cleavage of the H–O bond in the Volmer step, and (3) interaction with another H₂O molecule, followed by hydrogen gas evolution via either the Heyrovsky or Tafel pathways^31^. A critical descriptor of HER activity is the Gibbs free energy of hydrogen adsorption (∆G_H_), which ideally should be close to zero to ensure optimal catalytic performance. The adsorption energy (*E*_*ad*_), Gibbs free energy (∆G), and overpotential (η) were calculated using the following equations:

1 $$\:{E}_{ad}={E}_{srurf+H}-{E}_{surf}-\frac{1}{2}{E}_{{H}_{2}}$$

2 $$\:{\Delta\:}G={E}_{ad}+{\Delta\:}ZPE-T{\Delta\:}S$$

3 $$\eta\:\:=\varDelta\:G-E$$

where *E*_*surf*_, *E*_*surf*+*H*_, *E*_*H*2_, and *E* are the energies of clean, adsorbed surface with hydrogen atom and hydrogen molecule energy, and the standard equilibrium potentials (E = 0 for HER and 1.23 eV for OER), respectively. The difference between the change in zero-point energy denoted as *∆ZPE*, and the change in entropy, denoted as ∆S, multiplied by temperature T, among the gas phase and the adsorbed state, is equal to 0.24 eV. As illustrated in Fig. 3, C_1_, C_2_, C_3_, and h_1_ sites are the most favorable adsorption sites because they have the closest values to ∆*G* = 0^32^ with ∆*G* of 0.39, 0.25, 0.19, and − 0.32 eV, respectively. According to Eq. 3, the overpotential *η* is the same as *∆G* since E = 0 for HER, which indicates C_1_, C_2_, and C_3_ sites need extra potential to derive the reaction, while h_1_ site is more favorable than expected.

For comparative insight, the HER performance of other two-dimensional materials has also been explored. In particular, studies on 2D C₂₀ monolayers revealed an exceptionally favorable hydrogen adsorption site with a ∆G value of just 0.0147 eV, indicating near-optimal catalytic activity^33^. Additionally, Ti-, V-, Cr-, Mn-, Fe-, Co-, and Ni-decorated graphitic carbon nitride (g-C₃N₄) systems have been investigated, showing ΔG values ranging from − 0.02 to 0.81 eV for hydrogen adsorption on nitrogen atoms and from − 0.01 to 3.03 eV on transition metal (TM) atoms^34^. Among these configurations, Fe/g-C₃N₄ offered the most favorable site for hydrogen adsorption on nitrogen, while V/g-C₃N₄ exhibited the best adsorption characteristics on the TM atom.

### Oxygen evolution reaction (OER)

To gain a deeper understanding of the overall water splitting efficiency, the oxygen evolution reaction was investigated, comprising four sequential one-electron transfer steps^35,36^. Each step has a distinct Gibbs free energy change, which is crucial for evaluating the thermodynamic feasibility of the reaction. The four steps are:

4 $$\:{H}_{2}O\left(l\right)+\:*\:\to\:\:{OH}^{*}+{\text{H}}^{+}+{e}^{-}$$

5 $$\:{OH}^{*}\:\to\:\:{O}^{*}+{\text{H}}^{+}+{e}^{-}$$

6 $$\:{H}_{2}O\left(l\right)+\:{O}^{*}\:\to\:\:{OOH}^{*}+{\text{H}}^{+}+{e}^{-}$$

7 $$\:{\:OOH\:}^{*}\to\:\:*\:+{O}_{2}\left(g\right)+{H}^{+}+{e}^{-}$$

**Fig. 4:**
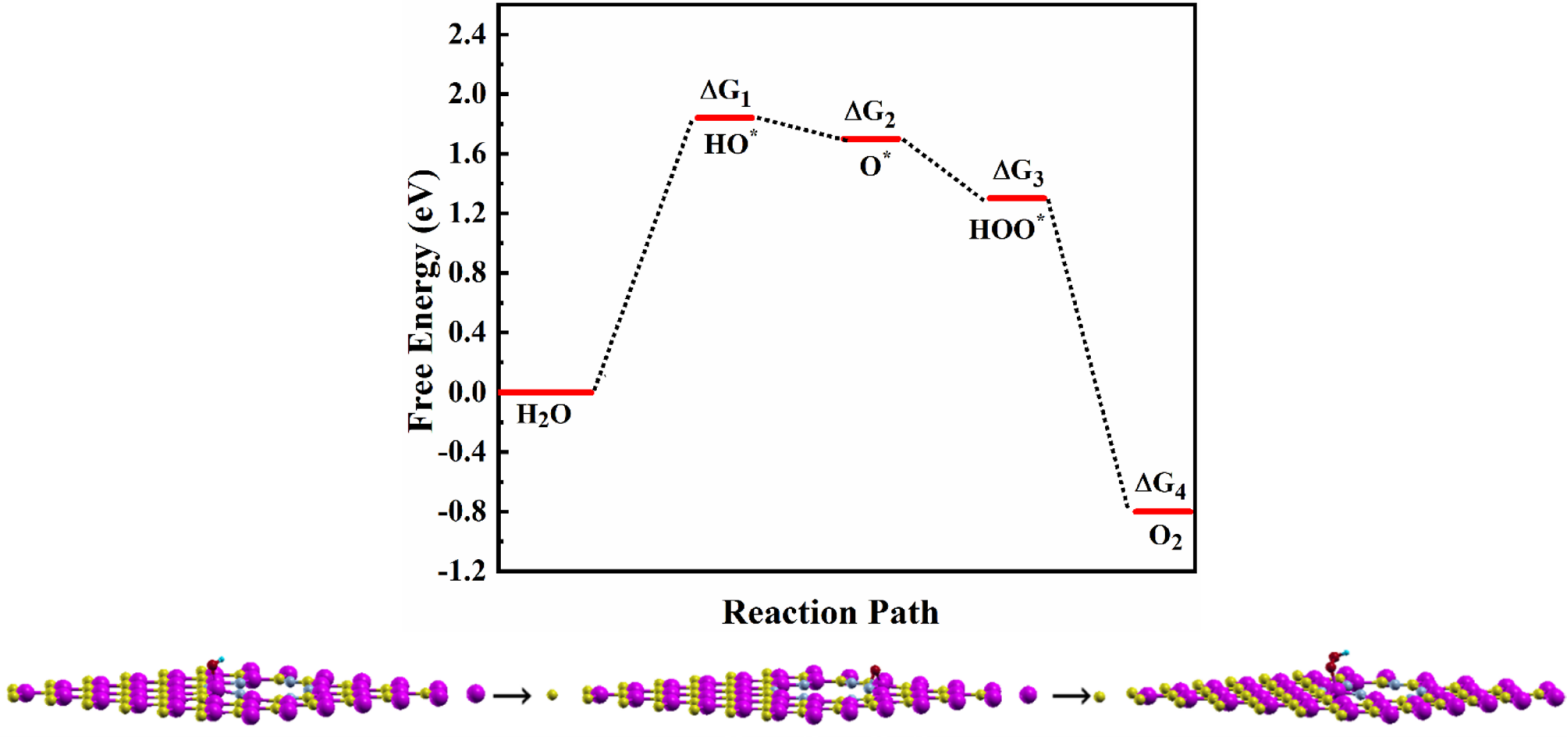
OER free energies of the intermediate states, calculated using DFT with the computational hydrogen electrode model.

In this context, * denotes the catalyst’s active site, *g* indicates the gas phase, and *O, *OOH, and *OH are the species adsorbed onto the catalyst’s active site. The conventional approach is commonly employed in literature to compute the reaction Gibbs free energy under standard conditions corresponding to Eqs. 7–10^37,38^.

8 $$\:{\Delta\:}{G}_{1}={E}_{HO}-{E}_{slab}+\frac{1}{2}{E}_{{H}_{2}}-{E}_{{H}_{2}O}+{\Delta\:}{E}_{ZPE}-T{\Delta\:}S$$

9 $$\:{\Delta\:}{\text{G}}_{2}={E}_{O}-{E}_{HO}+\frac{1}{2}{E}_{{H}_{2}}+{\Delta\:}{E}_{ZPE}-T{\Delta\:}S$$

10 $$\:{\Delta\:}{G}_{3}={E}_{HOO}-{E}_{O}+\frac{1}{2}{E}_{{H}_{2}}+{\Delta\:}{E}_{ZPE}-T{\Delta\:}S$$

11 $$\:{\Delta\:}{\text{G}}_{4}={E}_{slab}-{E}_{HOO}-\frac{3}{2}{E}_{{H}_{2}}+2{E}_{{H}_{2}O}+4.92+{\Delta\:}{E}_{ZPE}-T{\Delta\:}S$$

where *E*_*HO*_, *E*_*slab*_, *E*_*H*2_, *E*_*H*2*O*_, and *E*_*HOO*_ are the energies of the OH-adsorbed and bare structures, hydrogen molecule, water molecule, and HOO-adsorbed structure, respectively. To investigate the best adsorption site for OER, the adsorption energy for all available sites was calculated. Ge_1_ is found to be the most suitable candidate with an overpotential *η* = 0.61 V, indicating that an extra voltage beyond the minimum is required to derive the reaction. Figure 4 shows the free energies corresponding to Eqs. 7–10. The calculations indicate that ∆*G*_1_ corresponds to the rate-limiting step (RLS) in the OER. Specifically, the formation of OH is identified as the most energetically unfavorable step in the process, with a free energy of ∆*G*_1_ = 1.84 eV. Consequently, the energy barrier associated with OH formation is crucial in determining the efficiency of the OER, as it directly influences the rate at which the reaction can occur.

For the C₂₀ monolayer, the rate-limiting step of the oxygen evolution reaction (OER) was exhibited ΔG of 1.8127 eV^33^. To overcome this limitation, the formation of van der Waals (vdW) heterostructures has been explored by integrating C₂₀ with various materials to identify configurations capable of facilitating spontaneous OER. Notably, the GeS₂/C₂₀ heterostructure was shown to enable both HER and OER across a wide pH range, in contrast to pristine C₂₀, which supports only HER. Furthermore, Co/g-C₃N₄ and Ni/g-C₃N₄ systems demonstrate excellent OER activity, with low overpotentials of 0.61 V and 0.40 V, respectively, attributed to the moderate d-band center energies of the transition metal atoms^34^. Note that our much simpler material showed the same OER overpotential (0.61 V) of the complex Co/g-C₃N₄ and Ni/g-C₃N₄ systems, highlighting the potential of nitrogen-passivated germanium carbide nanomeshes as potential catalysts for photocatalytic water splitting.

**Fig. 5:**
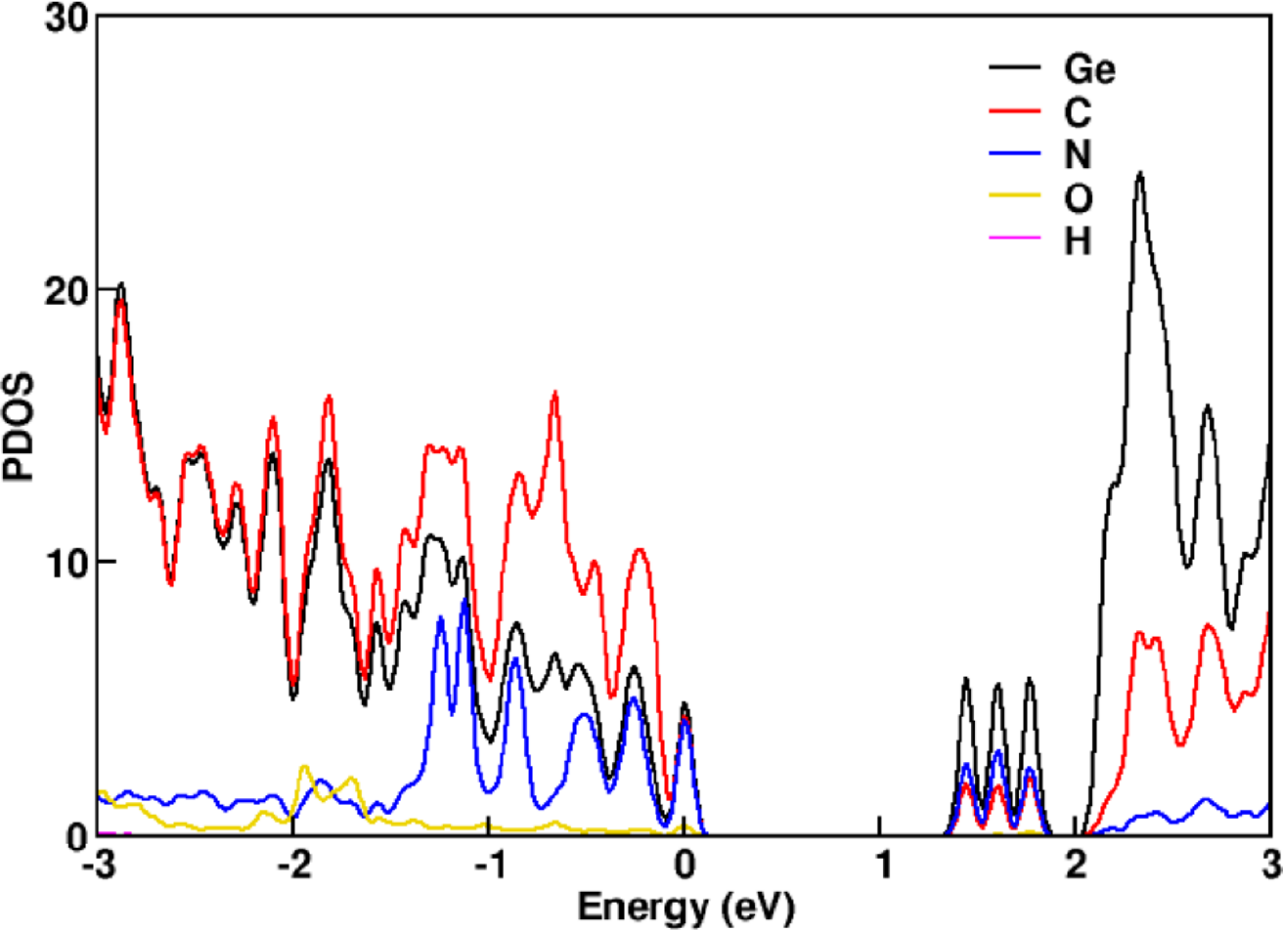
PDOS of OH-adsorbed N-passivated GeC nanomesh, showing contributions from Ge (black), C (red), N (blue), O (yellow), and H (pink) atoms.

**Fig. 6:**
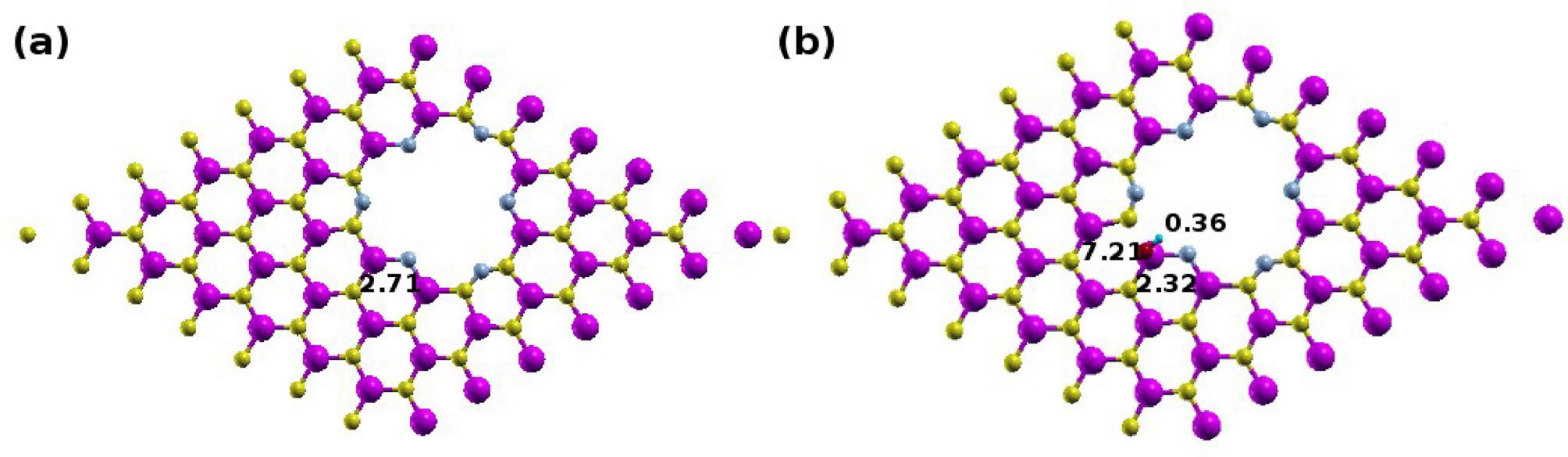
The Bader charge analysis for (**a**) bare and (**b**) OH-adsorbed N-passivated GeC nanomeshes, showing charge redistribution upon adsorption.

To further elucidate the behavior of OH adsorption on the N-passivated GeC nanomesh, PDOS and Bader charge analyses were performed. As illustrated in Fig. 5, the oxygen (O) atom contributes minimally to the valence band, while the hydrogen (H) atom shows an almost negligible contribution to the electronic states. This indicates that OH adsorption has little impact on the overall electronic structure of the material, particularly within the valence band region. Bader charge analysis (Fig. 6) confirms a net charge transfer from the OH molecule to the germanium (Ge) atom. Specifically, the Ge atom acquires approximately 0.39 |e|, resulting in a total charge of 2.32 |e|, while the O and H atoms carry charges of 7.23 |e| and 0.36 |e|, respectively.

**Fig. 7:**
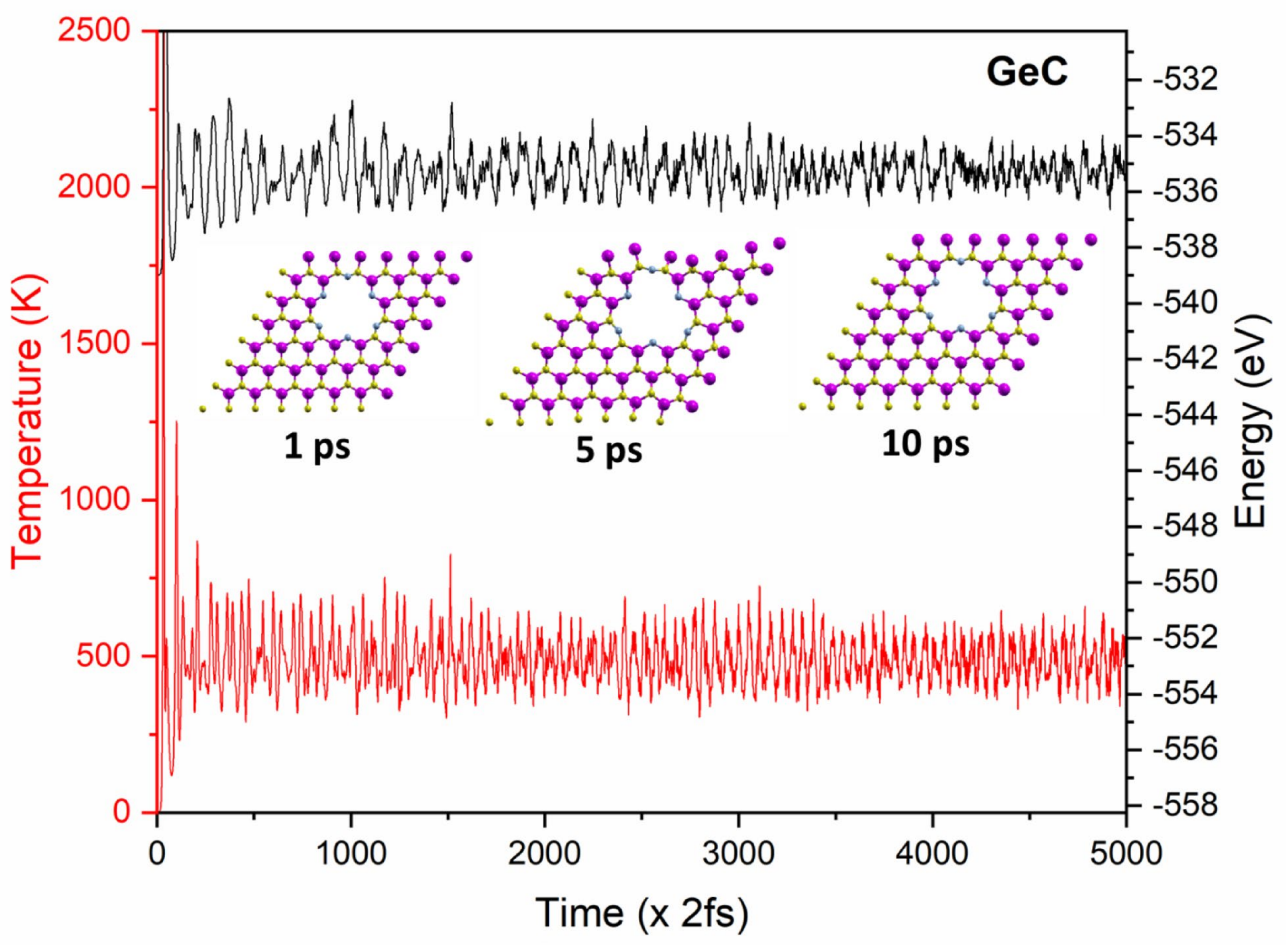
Temperature (red, left axis) and total energy (black, right axis) variations over time during the AIMD simulation of N-passivated GeC nanomesh at 500 K for 10 ps (5000 steps × 2 fs), including snapshots of the structure at 0 ps, 5 ps, and 10 ps.

Ab initio molecular dynamics (AIMD) simulations were performed to assess the catalyst’s stability, particularly its thermal resilience. The analysis was conducted at 500 K over a duration of 10 ps. As shown in Fig. 7, fluctuations in temperature and energy levels were observed throughout the simulations. However, the variations in total energy and temperature remained minimal, indicating that the catalyst maintains strong electrochemical and thermal stability. These findings confirm its suitability for experimental applications.

## Conclusion

In summary, this study demonstrates the potential of nitrogen-passivated GeC nanomeshes as efficient photocatalysts for overall water splitting. The introduction of periodic pores and nitrogen edge passivation effectively reduces the bandgap to 1.33 eV while positioning the valence and conduction band edges appropriately relative to the water oxidation and reduction potentials, respectively. These features satisfy the fundamental thermodynamic requirements for photocatalytic water splitting. Hydrogen evolution reaction (HER) analysis identified C_1_, C_2_, C_3_, and h_1_ as the most favorable adsorption sites, with ΔG values near thermoneutral, indicating efficient hydrogen adsorption and desorption kinetics. However, the oxygen evolution reaction (OER) remains a limiting step, with OH formation identified as the rate-determining step (ΔG₁ = 1.84 eV) with an overpotential of 0.61 V. Bader charge analysis revealed significant electron transfer from the OH adsorbate to the Ge atom, emphasizing the role of Ge sites in modulating surface reactivity. Overall, the nitrogen-passivated GeC nanomesh combines a suitable electronic structure with favorable HER activity, making it a promising 2D photocatalyst. However, overcoming the relatively high energy barrier associated with OER remains a challenge. Future work should explore co-catalyst integration, heterostructure engineering, or alternative surface functionalization strategies to further enhance OER activity and realize the full potential of this material for sustainable hydrogen production.

## Data Availability

The datasets used and/or analysed during the current study available from the corresponding author on reasonable request.
